# Environmentally persistent free radicals amplify ultrafine particle mediated cellular oxidative stress and cytotoxicity

**DOI:** 10.1186/1743-8977-6-11

**Published:** 2009-04-17

**Authors:** Shrilatha Balakrishna, Slawo Lomnicki, Kevin M McAvey, Richard B Cole, Barry Dellinger, Stephania A Cormier

**Affiliations:** 1 Department of Pharmacology and Experimental Therapeutics, Louisiana State University Health Sciences Center, New Orleans, Louisiana, USA; 2 Department of Chemistry, Louisiana State University, Baton Rouge, Louisiana, USA; 3 Department of Chemistry, University of New Orleans, New Orleans, Louisiana, USA; 4 Department of Pharmacology and Experimental Therapeutics, Louisiana State University Health Sciences Center, New Orleans, Louisiana, USA

## Abstract

**Background:**

Combustion generated particulate matter is deposited in the respiratory tract and pose a hazard to the lungs through their potential to cause oxidative stress and inflammation. We have previously shown that combustion of fuels and chlorinated hydrocarbons produce semiquinone-type radicals that are stabilized on particle surfaces (i.e. environmentally persistent free radicals; EPFRs). Because the composition and properties of actual combustion-generated particles are complex, heterogeneous in origin, and vary from day-to-day, we have chosen to use surrogate particle systems. In particular, we have chosen to use the radical of 2-monochlorophenol (MCP230) as the EPFR because we have previously shown that it forms a EPFR on Cu(II)O surfaces and catalyzes formation of PCDD/F. To understand the physicochemical properties responsible for the adverse pulmonary effects of combustion by-products, we have exposed human bronchial epithelial cells (BEAS-2B) to MCP230 or the CuO/silica substrate. Our general hypothesis was that the EPFR-containing particle would have greater toxicity than the substrate species.

**Results:**

Exposure of BEAS-2B cells to our combustion generated particle systems significantly increased reactive oxygen species (ROS) generation and decreased cellular antioxidants resulting in cell death. Resveratrol treatment reversed the decline in cellular glutathione (GSH), glutathione peroxidase (GPx), and superoxide dismutase (SOD) levels for both types of combustion-generated particle systems.

**Conclusion:**

The enhanced cytotoxicity upon exposure to MCP230 correlated with its ability to generate more cellular oxidative stress and concurrently reduce the antioxidant defenses of the epithelial cells (i.e. reduced GSH, SOD activity, and GPx). The EPFRs in MCP230 also seem to be of greater biological concern due to their ability to induce lipid peroxidation. These results are consistent with the oxidizing nature of the CuO/silica ultrafine particles and the reducing nature and prolonged environmental and biological lifetimes of the EPFRs in MCP230.

## Introduction

There is an increased focus on the potential adverse health effects associated with exposure to particulate matter (PM) including the development of cardiovascular diseases, pulmonary disease and cancer [1,2]. Exposure to PM can trigger respiratory distress in individuals with sensitive airways, and those at greater risk are the elderly and those with pre-existing respiratory or heart disease [3].

Ambient air contains a heterogeneous mixture of pollutants arising from various sources. A predominant source of ambient air pollution is emissions from combustion and/or thermal processes. The type and amount of combustion by-products generated depends on both the fuel type and the combustion appliance and process. Emissions from these processes include fine and ultrafine particles, NOx, VOCs, and other toxic products of incomplete combustion [4,5]. The particles contain organic carbon, metals, and other inorganic species. Interestingly, formation of chlorinated organic pollutants such as polychlorinated dibenzo-p-dioxin and polychlorinated dibenzofurans (PCDD/F) is associated with almost any combustion process in which chlorine and a transition metal are present. Chlorinated organic compounds are known to exhibit toxicity in both humans and animals [6-9]. Nevertheless, the health impacts and ramifications of exposure to combustion by-products are scarcely known.

Previous studies have demonstrated that PM_2.5 _(PM with a mean aerodynamic diameter of ≤ 2.5 μm) collected from six different cities across the country possess large quantities of radicals with chemical and toxicological characteristics similar to radicals of semiquinones [10]. It was subsequently demonstrated that very long lived (lifetimes on the order of an hour) semiquinone radicals are formed from molecular species that are adsorbed onto particle surfaces [11-13]. The radicals are formed via reaction with transition metal oxides that can be easily reduced by a chemisorbed organic compound converting the metal to a lower oxidation state [14]. During this process, an organic surface-bound radical is formed [11]. Association of the thus-formed free radical with the surface of the metal-containing particle stabilizes the radical [11,12]. This oxidized radical is in a dynamic equilibrium with the reduced form, with enough oxidized, free radical properties to be detectable by EPR spectroscopy, and enough reduced, chemisorbate properties to be stable and non-reactive. Radicals thus formed with a combination of stability and non-reactivity can be referred to as environmentally persistent or simply 'persistent' and can consequently be called 'environmentally persistent free radicals' (EPFRs) [2].

In a combustion system, the metals will predominantly exit the system at the highest oxidation state oxide. Iron, Nickel, Copper and Zinc are typically the highest concentration metals in combustion-generated particles with iron concentrations in the range of 0.1–1% and the other metals typically being present up to a few hundred ppm [14-16]. Transition metals such as copper are present in relatively high concentrations in biomass (woody wastes and debris) [17] and cigarette smoke and is a component of CCA (copper, chromate, arsenic) treated wood [18-20]. Cu(II)O is documented to catalyze PCDD/F formation through the chemisorption of simple precursors such as halogenated benzenes that form surface stabilized PFRs [12].

It has been proposed that molecular quinones take part in reactive oxygen species (ROS) generating cycles. In these cycles, quinones and hydroquinones, reduce molecular oxygen to superoxide in a process that generates semiquinone radicals as intermediates [11,21]. The superoxide reacts to form hydrogen peroxide which proceeds to form hydroxyl radical by the Fenton reaction with endogenous and possibly exogenous Fe^2+^. At issue in this mechanism is whether quinones exist in high enough concentrations and react fast enough to contribute to oxidative stress.

**Figure 1:**
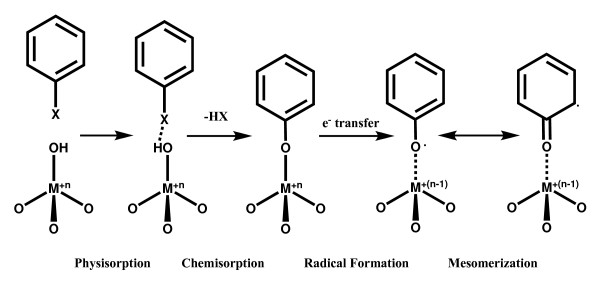
**Mechanism of formation of a phenoxyl-type PFR from a substituted aromatic on a metal oxide surface**. PFR formation proceeds through a mechanism of: 1) physisorption, 2) chemisorption by elimination of HX, and electron transfer to form the surface-associated PFR and a reduced metal. The resulting radical may be primarily oxygen-centered or carbon-centered based on the properties of the PFR-metal complex.

We hypothesize that the EPFRs associated with reduced metal oxides interact synergistically to produce ROS (superoxide, hydrogen peroxide, and hydroxyl radicals) while regenerating the EPFR and the oxidized form of the transition metal such that a true catalytic cycle occurs (Figure 1). Because actual airborne and combustion-generated particles contain a myriad of potentially toxic organic compounds and metal ions, we have chosen to use surrogate particle-EPFR systems. The particle substrate is silica, which is chemically inert. The transition metal oxide in our surrogate radical-particle systems is Cu(II)O (at 5%) because it is known to mediate the formation of EPFRs and PCDD/F, and it can be the dominant metal in combustion of biomass. The rationale of choosing the radical of 2-monochlorophenol was that it forms an EPFR on Cu(II)O surfaces and is known to catalyze formation of PCDD/F. The CuO/silica/2-MCP radical is referred to as MCP230 throughout the manuscript as it is formed by chemisorption on the CuO/silica particle at 230°C.

The objective in this study is to understand the potential effects of the EPFR-metal oxide particles on human bronchial epithelial cells. These studies shed light in understanding the toxicity profile associated with EPFRs as compared to that of simply an ultrafine particulate.

## Results

### Electron paramagnetic resonance of ultrafine particles

**Figure 2:**
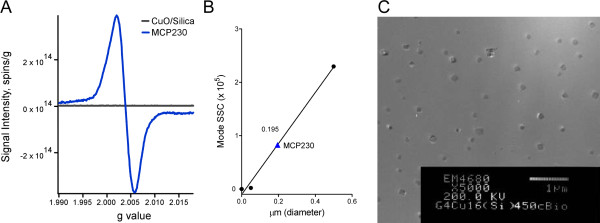
**Physical properties of ultrafine particles**: (**A**) EPR spectra of CuO/Silica and CuO/Silica exposed to 2-monochlorophenol at 230°C (MCP230). (**B**) Data showing the calculated size of MCP230 by flow cytometry. (**C**) Transmission electron micrograph of 100–200 nm Cab-o-sil Silica particles, containing CuO nanoclusters in an isotonic saline solution containing 0.02% tween-80. The procedure followed in preparation of this particle suspension is exactly the same in all studies presented in this manuscript. This figure demonstrates that the particles exist as singlets without aggregation.

The EPR measurement of the CuO/silica samples revealed the absence of signal that could be attributed to the presence of paramagnetic center (Figure 2A). This was not the case for the CuO/silica system exposed to MCP at 230°C (i.e. MCP230). In contrast, a strong signal appeared with a ΔH_p-p _width of 20 Gauss (Figure 2A). This signal could be deconvoluted mathematically into two components, one with a low g-value of ~2.002 and the other with a g-value equal to ~2.005. These two paramagnetic centers have been previously shown to be formed as a result of electron transfer between the chemisorbed 2-MCP molecule and copper center which results in formation of: 1) an F center in the copper oxide matrix (trapped electron in oxygen vacancy) and 2) a 2-chlorophenoxy radical [11,13]. The bidentate, chemisorbed radical previously reported to be formed at higher temperatures is not present, or is only present at very low concentrations, in the MCP230 samples. These radicals are stabilized by the metal center and are resistant to oxidation, decomposition, and recombination for a prolonged period.

### TEM and flow-cytometry of ultrafine particles

The properties of the ultrafine particles employed in our experiments are depicted in Figure 2. Aliquots of suspended weighed samples of MCP230 in saline + 0.02% Tween 80 were analyzed to evaluate their sizes by a flow cytometry. These results confirmed that the particles had a diameter of < 0.195 μm (Figure 2B). The transmission electron micrograph shown in Figure 2C further demonstrates that the particles, once suspended, are singlet in nature with little-to-no aggregation. Independent inductively coupled plasma atomic emission spectroscopy measurements of dissolved particles (dissolution accomplished by acidification in nitric acid) determined that the copper concentrations in MCP230 and CuO/silica samples were 3.330 ppm ± 0.169 and 3.990 ppm ± 0.234: respectively.

### Viability of BEAS-2B decreased with ultrafine particle exposure

**Figure 3:**
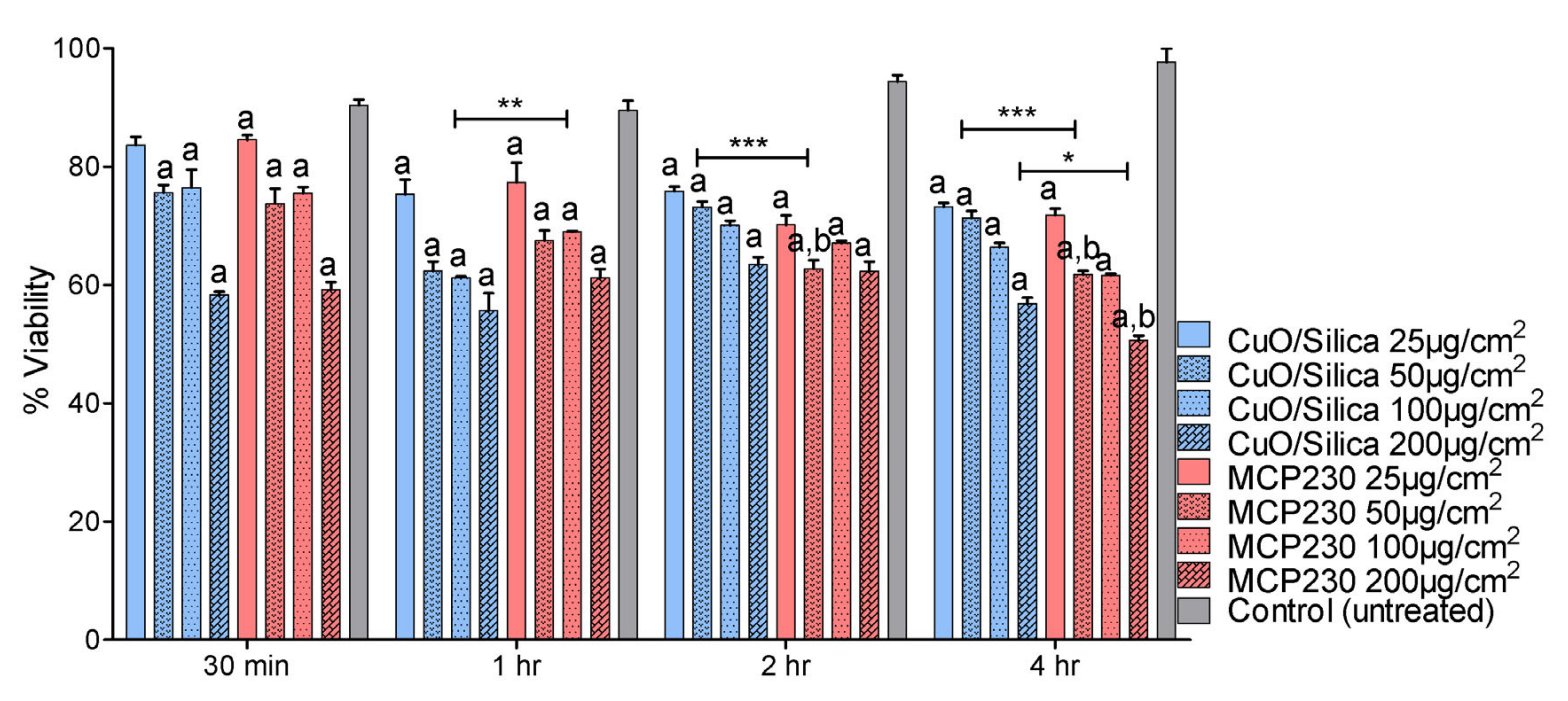
**Cytotoxicity in BEAS-2B cells exposed to CuO/Silica or MCP230 ultrafine particles for 0.5, 1, 2 and 4 h**. Results are expressed as mean ± SEM (n = 3). Significantly different from ^a^untreated controls or ^b^CuO/Silica at the same dose and exposure time (Two-way ANOVA; *P < 0.05, **P < 0.01, ***P < 0.001).

Time (2 – 4 h) and concentration (25 – 100 μg/cm^2^) dependent cytotoxicity assessments were performed with CuO/silica and MCP230 ultrafine particles. BEAS-2B cells were incubated with different concentrations (25, 50, 100 μg/cm^2^) of ultrafine particles (Figure 3) and their viability was determined at various time points. Cell viability decreased upon treatment with ultrafine particles in a time- and dose-dependent manner. Viability of the CuO/silica and MCP230 with a 100 μg/cm^2 ^concentration of ultrafine particles decreased on average to 68% of the control group at all exposure periods. Cells exposed to MCP230 exhibited 16% more cell death than those exposed to CuO/silica after 4 h of exposure.

### Ultrafine particles decreased the BEAS-2B cell-membrane integrity

**Figure 4:**
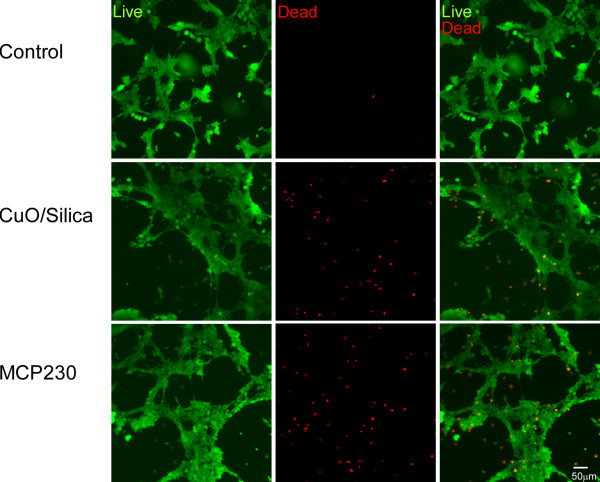
**Cell-membrane integrity of BEAS-2B cells on exposure to CuO/Silica and MCP230 ultrafine particles**. BEAS-2B cells were exposed for 4 h to 100 μg/cm^2 ^ultrafine particles. After the exposure, cells were treated with calcein AM and ethidium homodimer-1. Calcein AM enters all cells and is enzymatically converted to calcein. Cells with an intact plasma membrane (viable cells) retain calcein in the cytoplasm and thus fluoresce green. Cells with a compromised plasma membrane (dead cells) take up ethidium homodimer-1, which fluoresces red when complexed with nucleic acid. Scale bar = 50 μm.

To quantify live cell numbers based on the presence of their cytoplasmic membrane integrity, BEAS-2B cells were exposed to ultrafine particles. Calcein AM enters all cells, and is enzymatically converted to green-fluorescent Calcein in the cytoplasm. Cells with an intact plasma membrane (viable cells) retain Calcein, and thus fluoresce green. Only cells with a compromised plasma membrane (dead cells) take up ethidium homodimer-1. The red fluorescence of ethidium homodimer-1 is strongly enhanced once it interacts with the nucleic acids of the cell. Figure 4 shows BEAS-2B cell survival after 4 h of incubation with 100 μg/cm^2 ^of CuO/silica or MCP230 ultrafine particles. Alteration of cell membrane permeability was synchronized with loss of esterase activity. The percentage of cellular survival was as follows: Control, 97.706 ± 0.71%; CuO/silica, 66.311 ± 2.99%; MCP230, 61.325 ± 2.05%. Significant differences in cellular survival were observed between control and CuO/silica or MCP230 groups. There were no statistically significant differences between the CuO/silica and MCP230 treatment groups.

### Oxidative ability of ultrafine particles

**Figure 5:**
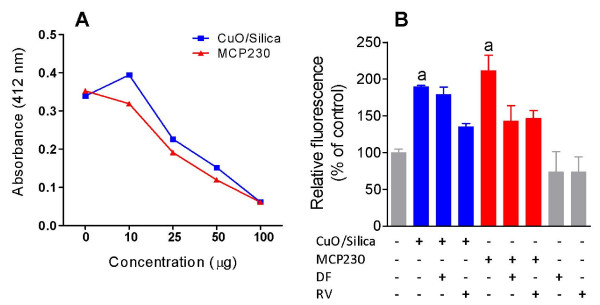
**(A) DTT activity with CuO/Silica, and MCP230 ultrafine particles at various concentrations**. (B) ROS generation in CuO/Silica or MCP230 ultrafine particles exposed BEAS-2B cells co-treated with 100 μM deferoxamine (DF) or 25 μM resveratrol (RV) for 4 h as measured by DCF assay. Results are expressed as mean ± SEM (n = 3). ^a ^significantly different from untreated controls.

Assessment of the oxidative ability of the ultrafine particles was determined using the DTT assay. MCP230 and CuO/silica ultrafine particles exhibited similar redox activity at the highest dose (50 μg) that was 8-fold greater than the silica ultrafine particles (Figure 5A). Regression analysis of the DTT assays exhibited a correlation coefficient (R^2^) of 0.933, 0.918 with CuO/silica and MCP230 respectively, suggesting the oxidative ability was incremental and progressive with the increase in the ultrafine particles concentration (data not shown).

The assay is typically interpreted in relation to the ability of quinones to oxidize DTT resulting in the formation of SQ radicals that then reduce O_2 _to O_2_^•-^, with concomitant formation of DTT-disulfide through the net reaction:



The actual assay measures the presence of DTT through its reaction with DTNB [24].

The reaction is logical for the MCP230 samples which we have previously shown to form SQ-type PFRs. However, it is impossible for SQ-type radicals to have a role in CuO/silica samples since quinones are not present. Since DTT is a strong reducing agent the likely reaction is:



not necessarily with concomitant formation of superoxide [26]. Since PM will contain large amounts of transition metals in their higher oxidation states, the use of the DTT assay with PM samples cannot be used to definitively indicate the formation of superoxide and other ROS as has been previously reported [24]. This assay must thus be interpreted against the background oxidation of DTT by the Cu(II)O/silica.

### Ultrafine particles induce the production of reactive oxygen species in vitro

Cleavage by intracellular esterases and subsequent oxidation of 2,7-dichlorofluorescin diacetate results in the formation of 2,7-dichlorofluorescin (DCF), which can be detected fluorometrically, and is thus an indicator of intracellular ROS production. Using the DCF assay, ultrafine particles were investigated for their stimulation of ROS formation during *in vitro *exposures of cultured human lung epithelial cells. Exposure of BEAS-2B cells to 100 μg/cm^2 ^of MCP230 and CuO/silica resulted in ~200% greater ROS levels (Figure 5B) when compared to untreated controls. Co-treatment with 25 μM resveratrol significantly alleviated (~70%) this increase in ROS levels (as indicated by the decrease in DCF fluorescence) in CuO/silica and MCP230 exposed cells; while co-treatment with 100 μM deferoxamine was only able to mitigate (69%) the increase in ROS levels in MCP230 exposed cells.

### Ultrafine particles perturb levels of intracellular glutathione and antioxidant enzymes

#### GSH assay

**Figure 6:**
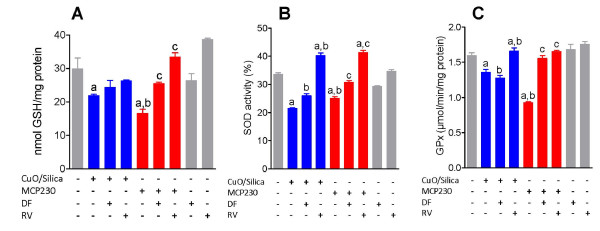
**Cellular antioxidant status of BEAS-2B cells exposed to CuO/Silica and MCP230 ultrafine particles**. Cells were co-treated with 100 μM deferoxamine (DF) or 25 μM resveratrol (RV) ± CuO/or MCP230 ultrafine particles for 4 h. (A) Depletion of intracellular glutathione (GSH) (B) Cytosolic superoxide dismutase (SOD) activity (C) Alterations in the glutathione peroxidase (GPx) enzyme activity. Results are expressed as mean ± SEM (n = 3). Significantly different from ^**a**^untreated controls; ^**b**^CuO/Silica; ^**c**^MCP230.

BEAS-2B cells were exposed to ultrafine particles to investigate whether they exert their effects on intracellular glutathione (GSH) levels. CuO/silica and MCP230 at concentrations of 100 μg/cm^2 ^decreased GSH levels by 26.7% and 45%, respectively (*p *< 0.05) at 4 h compared with the control values (Figure 6A). Co-treatment with 100 μM deferoxamine significantly increased the GSH levels of the MCP230 exposed cells by 65% and slightly elevated the levels of GSH in the CuO/silica exposed cells (30%, p = 0.24). 25 μM of resveratrol treatment completely reversed GSH depletion in MCP230 treated cells. CuO/silica exposed cells were protected by resveratrol to a lesser degree with elevated GSH levels 56% (p = 0.57).

#### SOD Assay

Cytosolic superoxide dismutase (SOD) activity was also significantly decreased in both CuO/silica and MCP230 exposed cells (36% and 25% respectively; Figure 6B). A statistically significant difference was observed between CuO/silica and MCP230 groups (P < 0.05). Treatment with deferoxamine moderately restored (~20%) the SOD levels in both the exposed groups; while resveratrol significantly reversed the depleted SOD levels in the CuO/silica and MCP230 treated cells.

#### GPx assay

In contrast to the other assays, response to the GPx assay was dominated by MCP230. A significant decrease (42.5%) in glutathione peroxidase (GPx) activity was observed in the cells exposed to MCP230 (Figure 6C) and slight decrease (16%) in the cells exposed to CuO/silica. Significant difference was also observed between CuO/silica and MCP230 groups (P < 0.05). Depleted levels of GPx were restored by treatment with both deferoxamine and resveratrol in the MCP230 treated cells, whereas GPx was restored only by resveratrol treatment in the CuO/silica treated cells.

### Ultrafine particles increased 8-isoprostane production in BEAS-2B cells

**Figure 7:**
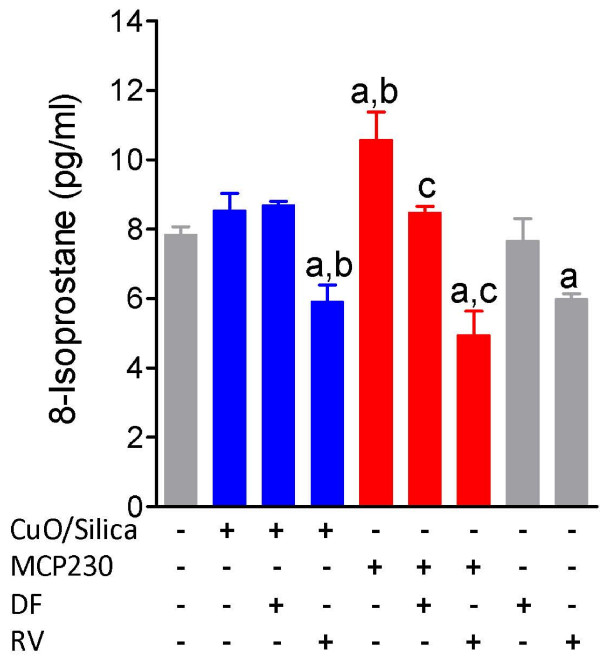
**Levels of by 8-isoprostane in BEAS -2B cell supernatant**. BEAS-2B cells were co-treated with 100 μM deferoxamine or 25 μM resveratrol and CuO/Silica or MCP230 ultrafine particles for 4 h in a 6-well plate with 1 ml culture media. Results are expressed as mean ± SEM. Significantly different from ^**a**^untreated controls; ^**b**^CuO/Silica; ^**c**^MCP230.

Isoprostanes are produced by the non-enzymatic random oxidation of cellular phospholipids by oxygen radicals and are considered ideal markers of oxidative stress. Analysis of culture supernatant from cells exposed to MCP230 ultrafine particles indicated significant increases in the levels of 8-isoprostane (Figure 7). Compared to non-treated cells, 8-isoprostane production increased (35%) in MCP230 exposed cells; whereas, there were no significant changes in the 8-isoprostane levels of CuO/silica treated cells. Likewise, a significant difference was observed between CuO/silica and MCP230 treated cells (P < 0.05). Co-treatment with resveratrol reduced the endogenous 8-isoprostane levels in MCP230 exposed cells by 24%. No significant change was evident with deferoxamine treatment compared to untreated particle exposed cells.

### Ultrafine particles stimulate inflammatory response

**Figure 8:**
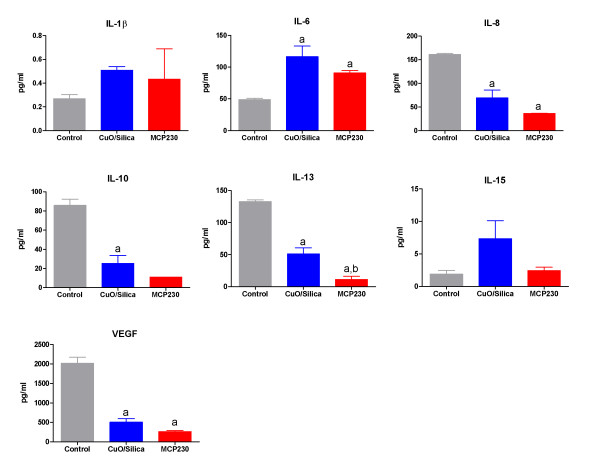
**Effect of CuO/Silica and MCP ultrafine particles on cytokine release from BEAS-2B at 4 h post-exposure**. Since IL-2, IL-12p40, IL12p70, IL-18, IFNγ, TNFα, IFNα2 were not detected at 4 h after ultrafine particle exposure, they were excluded from the figure. Results are expressed as mean ± SEM (n = 3). Significantly different from ^**a**^untreated controls, ^b^between CuO/Silica and MCP230 groups.

To characterize the degree of inflammatory changes induced by the ultrafine particle exposure, we analyzed the amount of various cytokines (IL-1β, IL-2, IL-6, IL-8, IL-10, IL-12p40, IL12p70, IL-13, IL-15, IL-18, IFNγ, TNFα, VEGF, IFNα2) in the cell-culture supernatants following exposure of CuO/silica and MCP230 for 4 h (Figure 8). IL-2, IL-12p40, IL12p70, IL-18, IFNγ, TNFα, and IFNα2 secretions were not significantly altered by exposure to either CuO/silica or MCP230 (data not shown). In contrast, IL-1β and IL-6 were secreted by BEAS-2B cells upon exposure to either CuO/silica or MCP230 exposure. Significant decreases in IL-8, IL-10, IL-13 and VEGF secretion were observed compared with untreated controls. Furthermore, levels of IL-13 were significantly different between the particle exposure groups.

## Discussion

ROS-induced oxidative stress is now recognized to be a prominent feature of many acute and chronic diseases and is currently considered to be one of the most significant causes of the health impacts of airborne particulate matter [11,2,35]. Semiquinone (SQ) radicals are a significant decomposition product resulting from the burning of biomass and coals [27-30] and have been demonstrated to be highly redox active and capable of producing ROS in biological systems [31,11,32]. [10]. Our results indicate that MCP230 has chemical and biological properties similar to that previously documented for SQ radicals [36]. Furthermore, the ability of metal oxide-containing, combustion-generated ultrafine particles, such as MCP230, to generate and stabilize SQ-type radicals on their surfaces suggests a previously unrecognized origin of the health effects attributed to ultrafine particles exposure. In particular, particle (surface)-induced stabilization of SQ-type radicals prolongs their environmental and biological lifetimes and enhances their potential for biological damage [37,38].

The MCP230 radical/particle system assessed in this study was formed by chemisorption of 2-MCP on a CuO/silica ultrafine particle. The formation is through the hydroxy-substituent of 2-MCP which formed primarily a 2-chlorophenoxyl radical [11] and reduced Cu^+2 ^to Cu^+1 ^at the site of chemisorption. Both 2-chlorophenoxyl and Cu^+1 ^are reducing agents that may act independently or synergistically to form ROS. Most of the Cu^+2 ^in the MCP230 samples was not converted to Cu^+1 ^and all of the copper in the CuO/Silica samples was considered to be Cu^+2^. Cu^+2^is an oxidizing agent that reacts with the substrates and other reactants in some of the assays. As stated earlier the DTT assay bears a significant error if transition metals are present in the particulate samples. Thus, the results for MCP230 in the DTT assay (where the particulate samples are still present) must be interpreted using the results of the assays on CuO/Silica as a control.

ROS can trigger a chain reaction on the cell membrane by oxidizing membrane phospholipids (a process called lipid peroxidation) and generate lipid hydroperoxide within the cell membrane [43]. Lipid peroxidation results in the formation of reactive aldehydes and isoprostanes (8-epi PF2α). 8-epi PF2α are ROS-catalyzed isomers of arachidonic acid and are stable lipid peroxidation products [44]. It is because of their relative stability *in vivo *that they have been used as markers of oxidative stress in both respiratory diseases such as asthma and COPD [45]. However, one isoprostane member, 8-isoprostane, has also been shown to be a strong stimulant of smooth muscle contraction through triggering the thromboxane A2 receptor and leading to small airways constriction [46,47]. Analysis of 8-isoprostane levels between CuO/silica and MCP230 exposed cells demonstrated that only PFR-containing ultrafine particles (MCP230) were capable of increasing 8-isoprostane levels and further suggest that these particles may be of greater biological and physiological concern.

The relationship between the biological effects of non-EPFR and EPFR-containing ultrafine particles was further characterized by treatment with antioxidants. In the present study, we tested the ability of resveratrol (3, 4', 5-trihydroxy-trans-stilbene) to attenuate ultrafine particle induced cellular oxidative stress. Treatment of MCP230 exposed cells with resveratrol significantly decreased EPFR-induced ROS production, which was associated with increased levels of GSH comparable to untreated controls. It is plausible that resveratrol attenuates ultrafine particles-mediated depletion of GSH levels by increasing the biosynthesis of GSH and also by scavenging ultrafine particles-induced ROS [39].

Hydroxyl groups at 4' and 5 positions make resveratrol a potent free radical scavenger [40]. Hence, it is probable that resveratrol is quenching free radicals generated by the ultrafine particles under our experimental conditions. Our data corroborates the previous observations pertaining to the antioxidant properties of resveratrol and its ability to scavenge free radicals such as •OH and O_2_•^- ^[39,41]. Further, it also indicates that the MCP230 ultrafine particles are generating •OH or O_2_•^- ^radicals which alter cellular oxidant-antioxidant homeostasis.

Figure 6 depicts a decrease in SOD activity in cells exposed to MCP230. The depletion in SOD activity was restored by resveratrol treatment (*vide infra*). Furthermore, significant depletion in GPx levels were restored by resveratrol in MCP230 treated epithelial cells signifying the role of resveratrol in up-regulation of GSH levels. The finding of a significant correlation between cellular redox imbalance (GSH, SOD, GPx levels) and redox activity of the particles (DTT production) provides further evidence for the role of ROS generation in particle toxicity.

Intriguingly, deferoxamine consistently exhibited little-to-no cellular benefit in the various assay results. When it did partially reverse the cellular effects upon particle exposure (see results, figure 6), the effects were similar between MCP230 and CuO/silica. Since deferoxamine is a metal chelator, it would similarly affect MCP230 and CuO/Silca as they both contain the same metal. However, deferoxamine was more efficient at restoring GPx activity to baseline levels in MCP230 treated cells suggesting that MCP230 generates more O_2_•^- ^radicals and further that the higher levels of O_2_•^- ^radicals maybe responsible for the enhanced lipid peroxidation observed with MCP230 group. In contrast, treatment with resveratrol resulted in significant cellular benefit including increased cell viability, which was associated with enhanced levels of cellular antioxidants and decreased lipid peroxidation. Resveratrol is a polyphenol phytoalexin and numerous studies have demonstrated it's ability to scavenge radicals [41,42]. However, our data with resveratrol demonstrated an increase in the levels of GSH, GPx, and SOD levels suggesting that resveratrol in our system is also exhibiting antioxidant signaling properties similar to that observed by Kode et al. [39]

Our studies revealed that CuO/silica and MCP230 ultrafine particles influenced the expression of inflammatory cytokines from BEAS-2B cells. Pollutants, such as diesel exhaust particles, are known to modify antigen presentation by suppressing IL-10 and upregulating IL-1 production [48]. Oxidative stress mediates the release of the pro-inflammatory cytokines and increases antigen presentation because of IL-10 downregulation [49] leading to increased sensitivity and inflammation. It has been proposed that an oxidant-antioxidant imbalance alter the VEGF homeostasis resulting in epithelial cell injury [49]. Our data further demonstrated that exposure of cells to ultrafine particles resulted in diminished IL-10 and VEGF release. As with diesel exhaust particle exposure, we believe that the production of ROS by the ultrafine particle systems is responsible for this decrease in IL-10 and VEGF secretion, but further investigation, is required to determine and clarify this mechanism. Although not tested here, the release of IL-6 by the ultrafine particle exposed cells might be the cause of the increased cell-death among particle exposed cells [50]. Previous studies confirm the correlation between maximal IL-6 induction and cell-death in normal BEAS-2B cells treated with capsaicin [51]. The cytokine data adds a potential and exciting viewpoint to the mechanisms that may be involved in particle induced airway injury and inflammation.

Epithelial cells play an important role in initiating or maintaining local inflammation of the airways by the interaction with inhaled components. Taken together, this study shows that combustion generated ultrafine particles, which constitute an important airborne pollutant in the urban environment, modulate the function of epithelial cells by modulating the release of pro-inflammatory cytokines. It further suggests that compounds with redox potential maybe a significant source of the respiratory effects observed upon particulate exposure. Thus, the physicochemical properties of combustion generated non-EPFR and EPFR containing ultrafine particles on airway epithelial cells may impair the inflammatory response of the lung, incapacitate epithelial repair mechanisms, and lead to respiratory dysfunction or disease exacerbation.

In summary, MCP230 and CuO/silica ultrafine particles were capable of inducing cytotoxicity in bronchial epithelial cells with MCP230 being more toxic at longer exposure times and equivalent doses. The enhanced cytotoxicity upon exposure to MCP230 correlated with its ability to generate more cellular oxidative stress (simultaneous increase in indicators of ROS, reduction in the antioxidant defenses of the epithelial cells (i.e. reduced GSH, SOD activity, and GPx) and increase in lipid peroxidation). The EPFRs in MCP230 seem to be of greater biological concern due to their pro-oxidant property which triggers redox signaling, lipid peroxidation, and inflammatory cascades that are involved in particle-induced cell injury. These results are consistent with the oxidizing nature of the CuO/silica ultrafine particles and the reducing nature and prolonged environmental and biological lifetimes of the PFRs in MCP230. More research is needed to sort out the reactions of these two very different ultrafine particles and their biological impacts.

## Conclusion

Inhalation of airborne ultra-fine particles is a major route of exposure to toxic combustion by-products; therefore, data generated from these studies are pertinent to virtually any combustion/thermal source of air pollution. This study indicates that the EPFRs associated with PM may be a key factor in the health effects of the latter. However, because the formation of the EPFR is concomitant with the formation of the reduced metal oxide, the individual impacts of the EPFR and reduced metal cannot be fully separated and the health impacts should be considered to be due to the PFR-particle system. Further studies to understand their acute molecular toxicity are ongoing and as are studies to determine the pathophysiological issues associated with exposure to combustion-generated, ultra-fine radical-particle systems with valid extrapolation to human exposure scenarios.

## Materials and methods

### Reagents

Dithiothreitol (DTT), deferoxamine, 2',7'-dichlorodihydrofluorescein-diacetate (DCFDA), 5,5'- dithio-bis(2-nitrobenzoic acid) (DTNB), copper nitrate (copper (II) nitrate hemipentahydrate, 99+%), 2-monochlorophenol (Aldrich, 99+%) were obtained from Sigma (St Louis, MO). Resveratrol (Axxora, CA), glutathione and superoxide dismutase assay kits were obtained from Sigma (Sigma, MO). All organic solvents were of Fisher optima grade (Fisher Scientific, Hampton, NH). Cabosil^®^- amorphous fumed silica EH-5 (Cabot Corp.)

### Synthesis of Ultrafine Particles

Particles of 5% CuO supported on silica were prepared by impregnation of ultrafine silica powder (Cabosil^® ^– particle size <200 nm) with copper nitrate hemipentahydrate using the incipient wetness method followed by calcination). The silica powder was introduced into a 0.1 M solution of the copper nitrate. The samples were stirred for 24 h at room temperature and dried at 120°C for 12 h before calcination in air for 5 h at 450°C. The adsorbate chemical, 2-monochlorophenol was used without further purification.

**Figure 9:**
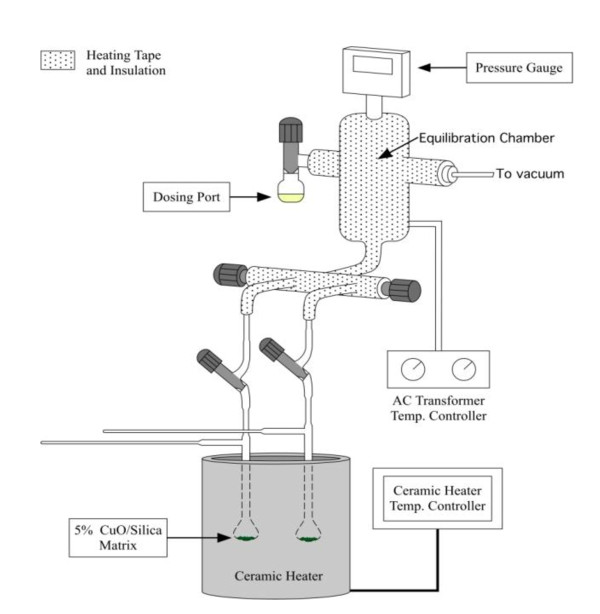
**Pictorial diagram of a two-port, particle dosing apparatus**.

The particulate samples were exposed to the vapors of the adsorbates using a custom made vacuum exposure system (Figure 9) that consists of a vacuum gauge, dosing vial port, equilibration chamber and 2 reactors. Each sample was re-oxidized *in situ *in air at 450°C and then evacuated to 10^-2 ^torr. The particles were dosed with adsorbate vapors at 10 torr at 230°C for 5 min. The port and dosing tube were evacuated for 1 h at the dosing temperature and 10^-2 ^torr to remove any residual physisorbed dosant. The reactor was then sealed under vacuum with a vacuum tight PFE stop-cock and allowed to cool to room temperature. The particle size was confirmed by transmission electron microscopy and flow cytometry.

### Measuring Electron paramagnetic resonance

Electron paramagnetic resonance (EPR) measurements were performed using a Bruker EMX-20/2.7 spectrometer at a microwave power of 1 mW, 9 GHz frequency, 4 G amplitude and 100 kHz frequency. The spectra were subjected to spectral de-convolution as non-derivative spectra using the Origin 7E Peak Fitting module, and the overall fit was compared with both the original absorption spectra and first derivative spectra.

### Flow Cytometry for Particle Sizing

For routine verification of particle size, flow cytometry was used. Flow cytometry was performed on a FACS Aria (BD Biosciences) using side scatter and fluorescent properties of 0.05 and 0.5 μm reference beads (Bangs Laboratories, IN) as previously described [22]. Flow data were analyzed and plotted using FlowJo software (Version 7.2.2 for windows, Tree Star, Inc).

### Transmission Electron Microscopy

Ultrafine particles (1 mg of 5% CuO/Cabosil) were added to 10 ml saline (with 0.02% Tween-80) and vortexed in a vial for 2 minutes. A drop of the resulting suspension was deposited on a TEM grid and the grid was air-dried. TEM was performed on a JEOL 2010 (JEOL Inc., Peabody, MA) with 200 keV electron energy.

### Inductively Coupled Plasma (ICP) – Atomic Emission Spectroscopy

The copper levels in MCP230 and CuO/SiO_2 _particles were measured by inductively coupled plasma – atomic emission spectroscopy using a Varian (Palo Alto, CA) Vista MPX spectrometer equipped with a charge coupled device (CCD) detector. Five characteristic emission wavelengths of copper were monitored and a minimum of 20 measurements were performed for each determination. Calibration standards were prepared by thoroughly dissolving purified copper powder (J.T. Baker Chemical Company, Phillipsburg, NJ) in a nitric acid solution (EMD Chemicals Inc., Gibbstown, NJ) whose final concentration was 4% nitric acid by volume.

### Culture of airway epithelial cells

BEAS-2B human bronchial epithelial cells [23] (Cat # CRL-9609; American Type Culture Collection, Manassas, VA) were used at passages 42–60. The cells were cultured in BEGM media (Lonza Walkersville Inc, MD, USA) containing growth supplements: human recombinant epidermal growth factor, hydrocortisone, insulin, bovine pituitary extract, ethanolamine, phosphoethanolamine, transferrin, 3,3'5-triiodothyronine, epinephrine, and retinoic acid. Culture flasks and multi-well plates were pre-coated with a mixture of 0.01 mg/ml fibronectin, 0.03 mg/ml bovine collagen type I and 0.01 mg/ml bovine serum albumin dissolved in LHC-9 medium. The cells were maintained in 75 cm^2 ^flasks at 37°C and 6% CO_2_. Media was replaced every 2 to 3 days, and cells were passaged when grown 85% confluent by dislodging with 0.25% trypsin-0.53 mM EDTA.

### Stimulation of airway epithelial cells

Cells grown to 80–90% confluence in 6-well polystyrene plates (Costar, Fisher Scientific) were exposed to media or 25–100 μg/cm^2 ^ultrafine particles in media (re-suspended by sonication and vortexing immediately before adding to the wells) followed by a co-treatment with 100 μM of deferoxamine or 25 μM of resveratrol for 4 hr. Positive controls were included to monitor changes in the BEAS-2B cell response. All experiments were replicated with at least two independent cell passages.

### Measurement of oxidative ability of ultrafine particles

The quantitative determination of ROS formation *in vitro *was made using the DTT assay [24] that enables estimation of the oxidative ability of the particles that can transfer electrons from DTT to oxygen. The loss of DTT is followed by its reaction with 5,5'-dithiobis-(2-nitrobenzoic acid) that is converted to 5-mercapto-2-nitrobenzoic. All samples were prepared at concentrations of 10, 25 and 50 μg/ml in 250 mM Tris-HCl buffer (pH 8.9). 20 μl of 16 mM DTT solution and 2 ml of a test sample, including a blank (250 mM Tris-HCl buffer only), were mixed in tubes and incubated for 10 min at 37°C in a water bath. 40 μl of 16 mM DTNB was added to this mixture. After the reaction was complete, 200 μl samples were placed in microtiter wells, and the absorbance was measured at 412 nm with a SpetraMax-M2 microplate reader (Molecular Devices, Sunnyvale, LA).

### Intracellular glutathione measurement

BEAS-2B cells grown to 85% confluence were treated with the particles for 4 hr. After the treatment, cells were washed with ice-cold PBS, scraped and lysed. Determination of reduced GSH was performed by using a commercial kit (Sigma, MO). The kit assay utilizes a thiol probe (monochlorobimane). When unbound, the probe shows very little fluorescence; however, when bound to reduced glutathione in a reaction that is catalyzed by glutathione S-transferase, it forms a strongly fluorescent adduct that can be measured using a microplate reader at excitation of 360 nm and emission wavelength of 485 nm.

### Antioxidant enzyme activity

The oxidation of glutathione is catalyzed by glutathione peoxidase (GPx). Oxidized glutathione is then reduced back to glutathione utilizing glutathione reductase and NADPH. The decrease in NADPH absorbance was measured at 340 nm during the oxidation of NADPH to NADP^+^. GPx activity was measured using a commercial kit from Sigma, MO. SOD activity was measured using the SOD assay kit (Sigma, MO) according to the manufacturer's instructions. After ultrafine particles exposure, cells were washed three times with cold PBS (pH 7.4) scraped into PBS, sonicated (three bursts of 50 W for 15 s; Sonics and Materials Inc, CT, USA) on ice, and lysates were centrifuged at 12,000 rpm for 20 min at 4°C and enzyme activities were assayed with the supernatants. Protein concentrations were determined by BCA assay (Thermo Fisher Scientific Inc., Waltham, MA)

### Measurement of intracellular reactive oxygen species

The production of reactive oxygen species was measured in BEAS-2B cells loaded with 10 μM 2,7-dichlorofluorescin diacetate at 37°C for 30 min in dark. The cells were then washed, incubated with 100 μM of deferoxamine or 25 μM of resveratrol and ultrafine particles in a 96-well plate for 4 hr. The increase in fluorescence was measured using a microplate reader with excitation at 485 nm and emission at 530 nm.

### Biomarker of lipid peroxidation (8-iso-PGF)

Lipid peroxidation was measured by a competitive enzyme-linked immunosorbent assay (ELISA) for 8-iso-PGF with a commercial kit (Cayman Chemical, Ann Arbor, MI). The assay is based on the competition between 8-iso-PGF and 8-isoprostane-acetylcholinestase (AChE) conjugate for a limited number of binding sites in each ELISA plate well. The concentration of 8-iso-PGF is inversely proportional to the number of binding sites available, whereas AChE is held constant. Samples (Culture supernatants) after the ultrafine particles exposure were transferred to the ELISA plate and incubated with the antibody for 18 hr. The absorbance of the colorimetric enzymatic reaction was read at 405 nm using the microplate reader and compared with an 8-iso-PGF standard curve to calculate concentration.

### Cytotoxicity

The effects of CuO/silica or MCP230 ultrafine particle treatments on the cytotoxicity of cells were determined by the Alamar blue assay [25], according to the manufacturer's protocol (Biosource, USA).

### Cell-membrane integrity assay

Cells grown on chambered slides were exposed to 100 μg/cm^2 ^of CuO/silica and MCP230 particle systems for 4 h. The cells were then incubated with 2 μM of calcein AM and 4 μM ethidium homodimer (Molecular probes, OR) for 30 – 45 minutes at room temperature. Cells were visualized using an inverted immunofluorescence microscope (Olympus, IX-70). Fluorescent images were obtained with a 10× objective using identical exposure times (2s) and images were acquired using a digital imaging software (SlideBook, Olympus). Cells with visible green fluorescence were scored as live; those with red fluorescence were scored as dead. Scale bar, 50 μm.

### Cytokine assays

Cell monolayers were exposed to CuO/silica or MCP230 ultrafine particles (100 μg/cm^2^) for 4 h. The cell-free supernatants were harvested at 4 h, and the presence of cytokines was determined using a high-throughput multiplex cytokine assay system (x-Plex human Assay; Bio-Rad) according to the manufacturer's instructions. Each sample was analyzed on the Bio-Plex 200 system (Bio-Rad). A broad sensitivity range of standards ranging from 1.95 to 4184.4 pg/ml (depending on the analyte) was used to quantitate a dynamic range of cytokine concentrations. The concentrations of analytes in these assays were quantified using a standard curve, and a nonlinear regression was performed to derive an equation that was then used to predict the concentration of the unknown samples. The following cytokines were assayed: IL-1β, IL-2, IL-6, IL-8, IL-10, IL-12p40, IL12p70, IL-13, IL-15, IL-18, IFNγ, TNFα, VEGF, IFNα2. Values below the range of sensitivity for the particular analyte were excluded. Since cell viability varied among exposures, results were expressed as pg/ml of % viable cells.

### Statistics

All data were plotted as mean ± SEM and analyzed using GraphPad Prism (GraphPad Software Inc., Version 5.0.0). One-way ANOVA was used to test for differences among the groups (Tukey's post-test). Two-way ANOVA was conducted for the cytotoxicity test (Bonferroni post-test). Differences were considered statistically significant if *p *< 0.05.

## Abbreviations

BEAS: human bronchial epithelium cells; DFX: deferoxamine; PCDD/F: polychlorinated dibenzo-p-dioxin and polychorniated dibenzofurans; EPFR: environmentally persistent free radicals; PM: particulate matter; ROS: reactive oxygen species; RV: resveratrol.

## Competing interests

The authors declare that they have no competing interests.

## Authors' contributions

SB and SC: Conceived the study, participated in its design and coordination and drafted the manuscript. SL and BD: Generation of ultrafine particles, EPR analysis. KM and RC: ICP analysis. All authors have read and revised the manuscript critically for important intellectual content and have given final approval of the version to be published.
